# Crystal structure of 5-{4′-[(2-{2-[2-(2-ammonio­eth­oxy)eth­oxy]eth­oxy}eth­yl)carbamo­yl]-4-meth­oxy-[1,1′-biphen­yl]-3-yl}-3-oxo-1,2,5-thia­diazo­lidin-2-ide 1,1-dioxide: a potential inhibitor of the enzyme protein tyrosine phosphatase 1B (PTP1B)

**DOI:** 10.1107/S2056989015003850

**Published:** 2015-03-04

**Authors:** Kasi Viswanatharaju Ruddraraju, Roman Hillebrand, Charles L. Barnes, Kent S. Gates

**Affiliations:** a 125 Chemistry Bldg, University of Missouri Columbia, MO 65211, USA

**Keywords:** crystal structure, PTP1B, inhibitor, 1,2,5-thia­diazo­lidin-3-one 1,1-dioxide, hydrogen bonding

## Abstract

The title compound, C_24_H_32_N_4_O_8_S, (I), crystallizes as a zwitterion. The terminal amine N atom of the [(2-{2-[2-(2-ammonio­eth­oxy)eth­oxy]eth­oxy}eth­yl)carbamo­yl] side chain is protonated, while the 1,2,5-thia­diazo­lidin-3-one 1,1-dioxide N atom is deprotonated. The side chain is turned over on itself with an intra­molecular N—H⋯O hydrogen bond. The 1,2,5-thia­diazo­lidin-3-one 1,1-dioxide ring has an envelope conformation with the aryl-substituted N atom as the flap. Its mean plane is inclined by 62.87 (8)° to the aryl ring to which it is attached, while the aryl rings of the biphenyl unit are inclined to one another by 20.81 (8)°. In the crystal, mol­ecules are linked by N—H⋯O and N—H⋯N hydrogen bonds, forming slabs lying parallel to (010). Within the slabs there are C—H⋯O and C—H⋯N hydrogen bonds and C—H⋯π inter­actions present.

## Abstract 2

A variety of 5-aryl-1,2,5-thia­diazo­lidin-3-one 1,1-dioxides have been developed as inhibitors of the enzyme protein tyrosine phosphatase 1B (PTP1B). For the title compound, there is the expected twisted relationship between the plane of the 1,2,5-thia­diazo­lidin-3-one 1,1-dioxide ring and the aryl ring to which it is attached, although the dihedral angle of 62.87 (8)° is substanti­ally less than that seen in certain protein–ligand structures.

## Chemical context   

**Figure 1:**
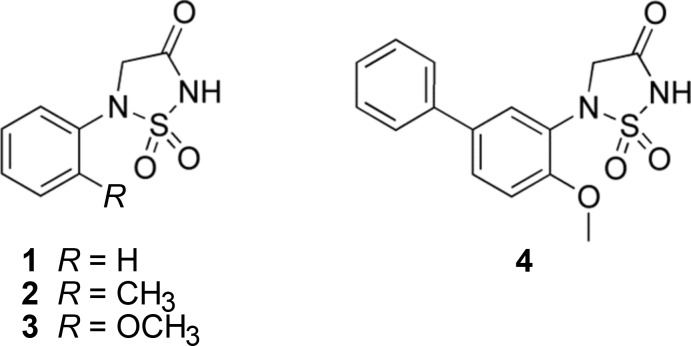
The parent compound **1** and related compounds.

A variety of 5-aryl-1,2,5-thia­diazo­lidin-3-one 1,1-dioxides have been developed as inhibitors of the enzyme protein tyrosine phosphatase 1B (PTP1B) (Combs, 2010 ▸). In this capacity, the 5-aryl-1,2,5-thia­diazo­lidin-3-one 1,1-dioxide core serves as a structural mimic of the phosphoryl tyrosine unit that is present in the endogenous substrates of the enzyme. The parent compound, 5-phenyl-1,2,5-thia­diazo­lidin-3-one 1,1-dioxide **1** (Fig. 1 ▸), is a rather weak inhibitor of PTP1B, displaying a K_i_ value of approximately 2 m*M* (Black *et al.*, 2005 ▸). Docking studies predicted that this compound must bind to the enzyme active site in a conformation where the planes of the 1,2,5-thia­diazo­lidin-3-one 1,1-dioxide and aryl rings are twisted, rather than co-planar (Black *et al.*, 2005 ▸). It was further anti­cipated that installation of substituents such as methyl or meth­oxy groups on the aryl ring at the position *ortho* to the 1,2,5-thia­diazo­lidin-3-one 1,1-dioxide substituent would bias the conformation of the free ligand toward the twisted form, thus serving to ‘pre-organize’ the compounds for binding to the enzyme active site (Black *et al.*, 2005 ▸). Indeed, compounds **2** and **3** (K_i_ values of 100 and 70 µ*M*, respectively**)** display substanti­ally higher affinities for PTP1B than does **1** (Black *et al.*, 2005 ▸). X-ray crystal structure analysis confirmed the twisted conformation of the 1,2,5-thia­diazo­lidin-3-one 1,1-dioxide and aryl ring systems in the protein–ligand co-crystal structure of **4** bound to PTP1B (Black *et al.*, 2005 ▸). The planes of these two rings are nearly perpendicular in the protein–ligand complex (dihedral angle of *ca* 88°, see: pdb code 2bgd). The ability of methyl and meth­oxy substit­uents to favor the twisted relationship between the 1,2,5-thia­diazo­lidin-3-one 1,1-dioxide and aryl rings in compounds like **2** and **3** has been studied computationally and the twisted relationship of these rings has been experimentally observed in the protein–ligand co-crystal structure of **4** with the enzyme PTP1B. However, to the best of our knowledge no crystal structures of free 5-aryl-1,2,5-thiadiazolidin-3-one 1,1-dioxides have been published. Herein, we describe the crystal structure of the title compound (I), shown in the scheme below, a derivative of compound **4**.
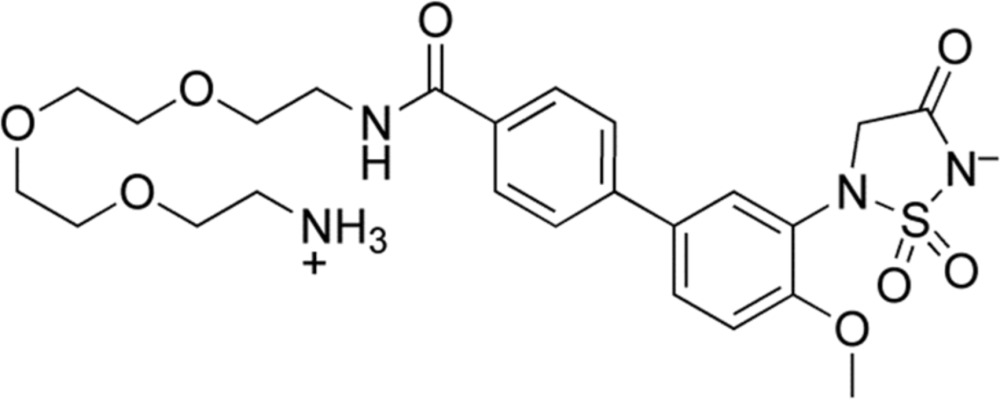


## Structural commentary   

**Figure 2:**
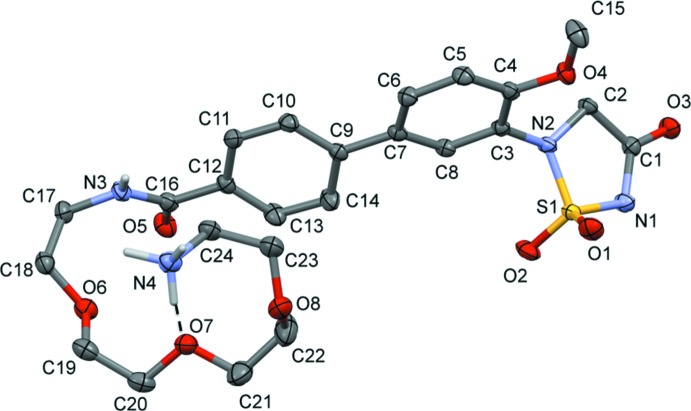
A view of the mol­ecular structure of the title compound (I), showing the atom labelling. Displacement ellipsoids are drawn at the 50% probability level. The intra­molecular N—H⋯O hydrogen bond is shown as a dashed line (see Table 1 ▸ for details) and C-bound H atoms have been omitted for clarity.

The title compound (I), crystallized as a zwitterion (Fig. 2 ▸). The terminal amine N atom, N4, is protonated and the 1,2,5-thia­diazo­lidin-3-one 1,1-dioxide nitro­gen atom, N1, is deprotonated. The [(2-{2-[2-(2-ammonio­eth­oxy)eth­oxy]eth­oxy}eth­yl)carbamo­yl] side chain is folded over on itself with an intra­molecular N—H⋯O hydrogen bond involving the ammonium group, N4, and an ether O atom, O7 (Table 1 ▸ and Fig. 2 ▸). The aryl rings of the biphenyl unit (C3–C8 and C9–C14) are inclined to one another by 20.81 (8)°. The 1,2,5-thia­diazo­lidin-3-one 1,1-dioxide ring (S1/N1/N2/C1/C2) has a shallow envelope conformation with nitro­gen atom N2 as the flap. Its mean plane is inclined to the benzene ring to which it is attached (C3–C8) by 62.87 (8)°. This twisted relationship between the planes of the 1,2,5-thia­diazo­lidin-3-one 1,1-dioxide and aryl rings is substanti­ally less than that seen in the protein–ligand co-crystal structure of **4** bound to PTP1B (Black *et al.*, 2005 ▸), where these two rings are nearly perpendicular to one another with a dihedral angle of *ca* 88° (see: Protein Data Bank entry: code 2bgd).

## Supra­molecular features   

**Figure 3:**
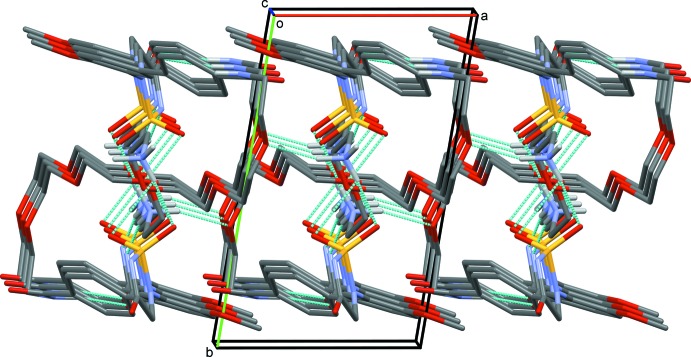
A view along the *c* axis of the crystal packing of the title compound. The N—H⋯O and N—H⋯O hydrogen bonds are shown as dashed lines (see Table 1 ▸ for details) and C-bound H atoms have been omitted for clarity.

In the crystal of (I), mol­ecules are linked by N—H⋯O and N—H⋯N hydrogen bonds, forming slabs lying parallel to the ac plane (Fig. 3 ▸ and Table 1 ▸). Within the slabs there are also C—H⋯O and C—H⋯N hydrogen bonds and C—H⋯π inter­actions present reinforcing the two-dimensional structure (Table 1 ▸).

## Database survey   

A search of the Cambridge Structural Database (Version 5.36; Groom & Allen, 2014 ▸) revealed no crystal structures of free 5-aryl-1,2,5-thia­diazo­lidin-3-one 1,1-dioxides. It did reveal the presence of five 1,2,5-thia­diazo­lidin-3-one 1,1-dioxide compounds substituted at the N atom in the 2-position. In the majority of these compounds, the five-membered 1,2,5-thia­diazo­lidine rings also have envelope conformations, with the N atom in the 5-position, as in compound (I), as the flap.

## Synthesis and crystallization   

The title compound was synthesized by amide bond formation between *tert*-butyl (2-{2-[2-(2-amino­eth­oxy)eth­oxy]eth­oxy}eth­yl)carbamate and 3′-(1,1-dioxido-4-oxo-1,2,5-thia­diazo­lidin-2-yl)-4′-meth­oxy-[1,1′-biphen­yl]-4-carb­oxy­lic acid *via* (benzotriazol-1-yl­oxy)tris­(di­methyl­amino)­phospho­nium hexa­fluoro­phosphate. The precursors were synthesized according to published procedures (Black *et al.*, 2005 ▸; Schwabacher *et al.*, 1998 ▸). Full synthetic details will be published elsewhere. Single crystals of the title compound (I) were obtained by slow evaporation of a solution of (I) in methanol.

## Refinement details   

Crystal data, data collection and structure refinement details are summarized in Table 2 ▸. The N-bound H atoms were located in a difference Fourier map and freely refined. The C-bound H atoms were included in calculated positions and treated as riding: C—H = 0.95–0.99 Å with *U*_iso_(H) = 1.5*U*_eq_(C) for methyl H atoms and = 1.2*U*_eq_(C) for other H atoms.

## Supplementary Material

Crystal structure: contains datablock(s) I, Global. DOI: 10.1107/S2056989015003850/su5087sup1.cif

Structure factors: contains datablock(s) I. DOI: 10.1107/S2056989015003850/su5087Isup2.hkl

Supporting information file. DOI: 10.1107/S2056989015003850/su5087Isup3.cml

CCDC reference: 1051176

Additional supporting information:  crystallographic information; 3D view; checkCIF report

## Figures and Tables

**Table 1:** Hydrogen-bond geometry (, ) *Cg*1 is the centroid of the C3C8 ring.

*D*H*A*	*D*H	H*A*	*D* *A*	*D*H*A*
N3H1*N*3O3^i^	0.82(2)	2.22(3)	3.012(2)	161(2)
N4H1*N*4O1^ii^	0.93(3)	2.29(3)	3.010(2)	133(2)
N4H1*N*4O7	0.93(3)	2.49(3)	3.106(2)	124(2)
N4H2*N*4N1^i^	1.03(3)	1.82(3)	2.821(2)	163(2)
N4H3*N*4O6^iii^	0.98(3)	1.99(3)	2.942(2)	162(3)
C2H2*B*O3^iv^	0.99	2.30	3.267(2)	166
C18H18*A*N1^i^	0.99	2.57	3.545(2)	168
C22H22*A*O8^ii^	0.99	2.63	3.343(3)	129
C24H24*A*O5^iii^	0.99	2.58	3.298(2)	129
C21H21*B* *Cg*1^ii^	0.99	2.70	3.555(2)	165

Symmetry codes: (i) 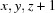; (ii) 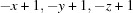; (iii) 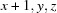; (iv) 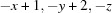.

**Table 2:** Experimental details

Crystal data	
Chemical formula	C_24_H_32_N_4_O_8_S
*M*_r_	536.59
Crystal system, space group	Triclinic, *P* 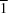
Temperature (K)	100
*a*, *b*, *c* ()	7.3483(2), 12.2233(3), 13.9847(4)
, , ()	95.323(1), 90.281(2), 99.802(1)
*V* (^3^)	1232.16(6)
*Z*	2
Radiation type	Cu *K*
(mm^1^)	1.67
Crystal size (mm)	0.15 0.15 0.02

Data collection	
Diffractometer	Bruker APEXII CCD area detector
Absorption correction	Multi-scan (*SADABS*; Bruker, 2008 ▸)
*T*_min_, *T*_max_	0.89, 0.97
No. of measured, independent and observed [*I* > 2(*I*)] reflections	15014, 4539, 4292
*R*_int_	0.017
(sin /)_max_ (^1^)	0.617

Refinement	
*R*[*F* ^2^ > 2(*F* ^2^)], *wR*(*F* ^2^), *S*	0.039, 0.111, 1.03
No. of reflections	4539
No. of parameters	351
H-atom treatment	H atoms treated by a mixture of independent and constrained refinement
_max_, _min_ (e ^3^)	0.56, 0.33

Computer programs: *APEX2* and *SAINT* (Bruker, 2008 ▸), *SHELXS2013* (Sheldrick, 2008 ▸), *SHELXL2013* (Sheldrick, 2015 ▸) and *Mercury* (Macrae *et al.*, 2008 ▸).

## supplementary crystallographic information

### Crystal data

**Table:**

C_24_H_32_N_4_O_8_S	*Z* = 2
*M**_r_* = 536.59	*F*(000) = 568
Triclinic, *P*1	*D*_x_ = 1.446 Mg m^−^^3^
*a* = 7.3483 (2) Å	Cu *K*α radiation, λ = 1.54178 Å
*b* = 12.2233 (3) Å	Cell parameters from 8971 reflections
*c* = 13.9847 (4) Å	θ = 3.2–71.7°
α = 95.323 (1)°	µ = 1.67 mm^−^^1^
β = 90.281 (2)°	*T* = 100 K
γ = 99.802 (1)°	Plate, colourless
*V* = 1232.16 (6) Å^3^	0.15 × 0.15 × 0.02 mm

### Data collection

**Table:**

Bruker APEXII CCD area-detector diffractometer	4292 reflections with *I* > 2σ(*I*)
Radiation source: Incoatec microfocus Cu tube	*R*_int_ = 0.017
ω and phi scans	θ_max_ = 72.1°, θ_min_ = 3.2°
Absorption correction: multi-scan (*SADABS*; Bruker, 2008)	*h* = −8→7
*T*_min_ = 0.89, *T*_max_ = 0.97	*k* = −15→15
15014 measured reflections	*l* = −16→16
4539 independent reflections	

### Refinement

**Table:**

Refinement on *F*^2^	0 restraints
Least-squares matrix: full	Hydrogen site location: mixed
*R*[*F*^2^ > 2σ(*F*^2^)] = 0.039	H atoms treated by a mixture of independent and constrained refinement
*wR*(*F*^2^) = 0.111	*w* = 1/[σ^2^(*F*_o_^2^) + (0.0647*P*)^2^ + 0.8661*P*] where *P* = (*F*_o_^2^ + 2*F*_c_^2^)/3
*S* = 1.03	(Δ/σ)_max_ < 0.001
4539 reflections	Δρ_max_ = 0.56 e Å^−^^3^
351 parameters	Δρ_min_ = −0.33 e Å^−^^3^

### Special details

**Table:**

Geometry. All e.s.d.'s (except the e.s.d. in the dihedral angle between two l.s. planes) are estimated using the full covariance matrix. The cell e.s.d.'s are taken into account individually in the estimation of e.s.d.'s in distances, angles and torsion angles; correlations between e.s.d.'s in cell parameters are only used when they are defined by crystal symmetry. An approximate (isotropic) treatment of cell e.s.d.'s is used for estimating e.s.d.'s involving l.s. planes.
Refinement. Maximum electron density of 0.56 e is in the vicinity of C21 in the extended chain and may represent very minor disorder.

### Fractional atomic coordinates and isotropic or equivalent isotropic displacement parameters (Å^2^)

**Table:**

	*x*	*y*	*z*	*U*_iso_*/*U*_eq_	
S1	0.52151 (5)	0.68948 (3)	0.09933 (3)	0.01945 (13)	
O1	0.65998 (19)	0.61868 (11)	0.09811 (10)	0.0305 (3)	
O2	0.35802 (18)	0.64660 (11)	0.14874 (10)	0.0317 (3)	
O3	0.52042 (18)	0.86988 (10)	−0.09372 (9)	0.0266 (3)	
O4	0.97456 (17)	0.88464 (10)	0.17601 (9)	0.0242 (3)	
O5	−0.07338 (17)	0.81837 (11)	0.69084 (9)	0.0296 (3)	
O6	−0.04449 (18)	0.63027 (11)	0.85611 (9)	0.0279 (3)	
O7	0.16486 (19)	0.49974 (10)	0.73225 (10)	0.0305 (3)	
O8	0.5123 (2)	0.49283 (11)	0.63370 (10)	0.0359 (3)	
N1	0.4761 (2)	0.71885 (12)	−0.00657 (10)	0.0239 (3)	
N2	0.6032 (2)	0.81729 (11)	0.14491 (10)	0.0221 (3)	
N3	0.1468 (2)	0.84118 (12)	0.80862 (11)	0.0232 (3)	
H1N3	0.256 (3)	0.8427 (18)	0.8228 (16)	0.027 (6)*	
N4	0.5520 (3)	0.58747 (14)	0.82658 (12)	0.0291 (3)	
H1N4	0.474 (4)	0.520 (3)	0.812 (2)	0.052 (8)*	
H2N4	0.510 (3)	0.621 (2)	0.8909 (19)	0.041 (6)*	
H3N4	0.685 (5)	0.585 (2)	0.831 (2)	0.058 (8)*	
C1	0.5371 (2)	0.82684 (14)	−0.01819 (12)	0.0206 (3)	
C2	0.6324 (2)	0.89346 (13)	0.07032 (11)	0.0198 (3)	
H2A	0.7659	0.9167	0.0595	0.024*	
H2B	0.5769	0.9607	0.0878	0.024*	
C3	0.6802 (2)	0.83838 (13)	0.23985 (12)	0.0187 (3)	
C4	0.8705 (2)	0.87285 (13)	0.25612 (12)	0.0204 (3)	
C5	0.9381 (2)	0.89174 (14)	0.35051 (13)	0.0229 (4)	
H5	1.0668	0.9150	0.3628	0.028*	
C6	0.8188 (2)	0.87689 (14)	0.42708 (12)	0.0224 (3)	
H6	0.8679	0.8896	0.4909	0.027*	
C7	0.6289 (2)	0.84384 (13)	0.41224 (12)	0.0193 (3)	
C8	0.5638 (2)	0.82427 (13)	0.31696 (12)	0.0197 (3)	
H8	0.4352	0.8005	0.3047	0.024*	
C9	0.4964 (2)	0.83521 (13)	0.49259 (12)	0.0195 (3)	
C10	0.5441 (2)	0.88953 (14)	0.58384 (12)	0.0223 (3)	
H10	0.6661	0.9293	0.5958	0.027*	
C11	0.4178 (2)	0.88685 (14)	0.65740 (12)	0.0230 (4)	
H11	0.4549	0.9235	0.7190	0.028*	
C12	0.2375 (2)	0.83098 (13)	0.64171 (12)	0.0204 (3)	
C13	0.1889 (3)	0.77461 (16)	0.55146 (13)	0.0282 (4)	
H13	0.0665	0.7354	0.5396	0.034*	
C14	0.3165 (3)	0.77503 (16)	0.47882 (13)	0.0281 (4)	
H14	0.2816	0.7338	0.4187	0.034*	
C15	1.1677 (3)	0.92460 (19)	0.18946 (15)	0.0334 (4)	
H15A	1.1870	0.9942	0.2319	0.050*	
H15B	1.2233	0.9383	0.1272	0.050*	
H15C	1.2255	0.8687	0.2185	0.050*	
C16	0.0893 (2)	0.82919 (14)	0.71575 (13)	0.0226 (4)	
C17	0.0153 (3)	0.82894 (15)	0.88628 (13)	0.0270 (4)	
H17A	−0.1093	0.8332	0.8612	0.032*	
H17B	0.0500	0.8914	0.9369	0.032*	
C18	0.0089 (3)	0.71993 (16)	0.92983 (13)	0.0273 (4)	
H18A	0.1319	0.7157	0.9570	0.033*	
H18B	−0.0813	0.7146	0.9822	0.033*	
C19	0.0108 (3)	0.52875 (16)	0.87786 (14)	0.0294 (4)	
H19A	−0.0770	0.4912	0.9229	0.035*	
H19B	0.1350	0.5449	0.9090	0.035*	
C20	0.0145 (3)	0.45443 (15)	0.78734 (15)	0.0303 (4)	
H20A	0.0284	0.3787	0.8026	0.036*	
H20B	−0.1026	0.4487	0.7505	0.036*	
C21	0.1833 (3)	0.42988 (19)	0.64803 (17)	0.0431 (5)	
H21A	0.0660	0.4151	0.6101	0.052*	
H21B	0.2117	0.3577	0.6651	0.052*	
C22	0.3357 (4)	0.4859 (2)	0.58984 (17)	0.0510 (6)	
H22A	0.3335	0.4440	0.5256	0.061*	
H22B	0.3141	0.5621	0.5807	0.061*	
C23	0.6108 (3)	0.60309 (16)	0.65657 (15)	0.0344 (4)	
H23A	0.5972	0.6482	0.6026	0.041*	
H23B	0.7437	0.6005	0.6651	0.041*	
C24	0.5416 (3)	0.65799 (15)	0.74680 (14)	0.0297 (4)	
H24A	0.6178	0.7324	0.7631	0.036*	
H24B	0.4123	0.6680	0.7366	0.036*	

### Atomic displacement parameters (Å^2^)

**Table:**

	*U*^11^	*U*^22^	*U*^33^	*U*^12^	*U*^13^	*U*^23^
S1	0.0198 (2)	0.0170 (2)	0.0207 (2)	−0.00143 (15)	−0.00151 (15)	0.00555 (14)
O1	0.0350 (8)	0.0252 (6)	0.0340 (7)	0.0097 (5)	0.0005 (6)	0.0085 (5)
O2	0.0250 (7)	0.0316 (7)	0.0353 (7)	−0.0080 (5)	0.0031 (6)	0.0098 (6)
O3	0.0334 (7)	0.0249 (6)	0.0221 (6)	0.0042 (5)	−0.0041 (5)	0.0077 (5)
O4	0.0183 (6)	0.0312 (6)	0.0231 (6)	0.0026 (5)	0.0036 (5)	0.0060 (5)
O5	0.0197 (7)	0.0391 (7)	0.0312 (7)	0.0057 (5)	0.0018 (5)	0.0076 (6)
O6	0.0264 (7)	0.0275 (6)	0.0308 (7)	0.0055 (5)	0.0002 (5)	0.0065 (5)
O7	0.0299 (7)	0.0246 (6)	0.0351 (7)	0.0004 (5)	0.0045 (6)	0.0007 (5)
O8	0.0422 (8)	0.0267 (7)	0.0368 (7)	0.0029 (6)	0.0066 (6)	−0.0014 (6)
N1	0.0289 (8)	0.0204 (7)	0.0217 (7)	0.0008 (6)	−0.0043 (6)	0.0044 (6)
N2	0.0275 (8)	0.0182 (7)	0.0188 (7)	−0.0036 (5)	−0.0031 (6)	0.0064 (5)
N3	0.0194 (8)	0.0262 (8)	0.0237 (7)	0.0021 (6)	0.0040 (6)	0.0032 (6)
N4	0.0342 (10)	0.0243 (8)	0.0300 (8)	0.0068 (7)	−0.0004 (7)	0.0049 (6)
C1	0.0199 (8)	0.0219 (8)	0.0206 (8)	0.0042 (6)	−0.0002 (6)	0.0039 (6)
C2	0.0218 (8)	0.0176 (7)	0.0201 (8)	0.0006 (6)	−0.0006 (6)	0.0069 (6)
C3	0.0212 (8)	0.0158 (7)	0.0188 (8)	0.0015 (6)	−0.0018 (6)	0.0042 (6)
C4	0.0214 (9)	0.0180 (7)	0.0226 (8)	0.0035 (6)	0.0029 (7)	0.0049 (6)
C5	0.0166 (8)	0.0252 (8)	0.0263 (9)	0.0009 (6)	−0.0020 (7)	0.0035 (7)
C6	0.0219 (9)	0.0237 (8)	0.0212 (8)	0.0023 (6)	−0.0033 (7)	0.0026 (6)
C7	0.0206 (9)	0.0174 (7)	0.0205 (8)	0.0031 (6)	0.0002 (6)	0.0050 (6)
C8	0.0176 (8)	0.0189 (8)	0.0225 (8)	0.0015 (6)	−0.0012 (6)	0.0051 (6)
C9	0.0202 (9)	0.0188 (8)	0.0207 (8)	0.0039 (6)	−0.0003 (6)	0.0065 (6)
C10	0.0200 (9)	0.0226 (8)	0.0230 (8)	−0.0006 (6)	−0.0004 (7)	0.0031 (6)
C11	0.0266 (9)	0.0205 (8)	0.0210 (8)	0.0014 (6)	0.0001 (7)	0.0019 (6)
C12	0.0216 (9)	0.0201 (8)	0.0212 (8)	0.0052 (6)	0.0012 (6)	0.0072 (6)
C13	0.0204 (9)	0.0373 (10)	0.0251 (9)	−0.0017 (7)	−0.0024 (7)	0.0052 (7)
C14	0.0251 (10)	0.0365 (10)	0.0202 (8)	−0.0020 (7)	−0.0017 (7)	0.0019 (7)
C15	0.0175 (9)	0.0517 (12)	0.0328 (10)	0.0062 (8)	0.0031 (8)	0.0133 (9)
C16	0.0229 (10)	0.0196 (8)	0.0258 (9)	0.0033 (6)	0.0030 (7)	0.0053 (6)
C17	0.0255 (9)	0.0293 (9)	0.0252 (9)	0.0032 (7)	0.0081 (7)	0.0006 (7)
C18	0.0241 (9)	0.0340 (10)	0.0231 (8)	0.0022 (7)	0.0040 (7)	0.0038 (7)
C19	0.0231 (9)	0.0292 (9)	0.0367 (10)	0.0016 (7)	−0.0006 (8)	0.0131 (8)
C20	0.0226 (9)	0.0249 (9)	0.0438 (11)	0.0018 (7)	0.0007 (8)	0.0094 (8)
C21	0.0386 (12)	0.0417 (12)	0.0427 (12)	−0.0026 (9)	0.0010 (10)	−0.0108 (10)
C22	0.0471 (14)	0.0703 (17)	0.0295 (11)	−0.0007 (12)	0.0019 (10)	−0.0079 (10)
C23	0.0421 (12)	0.0263 (9)	0.0336 (10)	0.0014 (8)	0.0054 (9)	0.0049 (8)
C24	0.0369 (11)	0.0225 (9)	0.0296 (9)	0.0024 (7)	0.0016 (8)	0.0065 (7)

### Geometric parameters (Å, º)

**Table:**

S1—O2	1.4341 (13)	C8—H8	0.9500
S1—O1	1.4429 (13)	C9—C10	1.397 (2)
S1—N1	1.6025 (14)	C9—C14	1.402 (3)
S1—N2	1.6429 (14)	C10—C11	1.388 (2)
O3—C1	1.237 (2)	C10—H10	0.9500
O4—C4	1.365 (2)	C11—C12	1.390 (2)
O4—C15	1.425 (2)	C11—H11	0.9500
O5—C16	1.226 (2)	C12—C13	1.395 (3)
O6—C19	1.428 (2)	C12—C16	1.505 (2)
O6—C18	1.435 (2)	C13—C14	1.386 (3)
O7—C21	1.410 (2)	C13—H13	0.9500
O7—C20	1.414 (2)	C14—H14	0.9500
O8—C22	1.419 (3)	C15—H15A	0.9800
O8—C23	1.424 (2)	C15—H15B	0.9800
N1—C1	1.345 (2)	C15—H15C	0.9800
N2—C3	1.425 (2)	C17—C18	1.509 (3)
N2—C2	1.454 (2)	C17—H17A	0.9900
N3—C16	1.351 (2)	C17—H17B	0.9900
N3—C17	1.459 (2)	C18—H18A	0.9900
N3—H1N3	0.82 (2)	C18—H18B	0.9900
N4—C24	1.481 (2)	C19—C20	1.491 (3)
N4—H1N4	0.93 (3)	C19—H19A	0.9900
N4—H2N4	1.03 (3)	C19—H19B	0.9900
N4—H3N4	0.98 (3)	C20—H20A	0.9900
C1—C2	1.515 (2)	C20—H20B	0.9900
C2—H2A	0.9900	C21—C22	1.496 (3)
C2—H2B	0.9900	C21—H21A	0.9900
C3—C8	1.385 (2)	C21—H21B	0.9900
C3—C4	1.401 (2)	C22—H22A	0.9900
C4—C5	1.393 (2)	C22—H22B	0.9900
C5—C6	1.393 (2)	C23—C24	1.505 (3)
C5—H5	0.9500	C23—H23A	0.9900
C6—C7	1.394 (2)	C23—H23B	0.9900
C6—H6	0.9500	C24—H24A	0.9900
C7—C8	1.399 (2)	C24—H24B	0.9900
C7—C9	1.490 (2)		
			
O2—S1—O1	113.21 (8)	C14—C13—H13	119.5
O2—S1—N1	112.24 (8)	C12—C13—H13	119.5
O1—S1—N1	111.49 (8)	C13—C14—C9	121.12 (17)
O2—S1—N2	109.69 (8)	C13—C14—H14	119.4
O1—S1—N2	111.90 (8)	C9—C14—H14	119.4
N1—S1—N2	97.26 (7)	O4—C15—H15A	109.5
C4—O4—C15	117.63 (14)	O4—C15—H15B	109.5
C19—O6—C18	112.83 (14)	H15A—C15—H15B	109.5
C21—O7—C20	111.72 (15)	O4—C15—H15C	109.5
C22—O8—C23	115.10 (18)	H15A—C15—H15C	109.5
C1—N1—S1	111.85 (12)	H15B—C15—H15C	109.5
C3—N2—C2	125.75 (13)	O5—C16—N3	123.27 (16)
C3—N2—S1	120.81 (11)	O5—C16—C12	120.31 (16)
C2—N2—S1	111.22 (11)	N3—C16—C12	116.41 (16)
C16—N3—C17	121.27 (16)	N3—C17—C18	112.19 (15)
C16—N3—H1N3	120.7 (16)	N3—C17—H17A	109.2
C17—N3—H1N3	116.9 (15)	C18—C17—H17A	109.2
C24—N4—H1N4	108.5 (17)	N3—C17—H17B	109.2
C24—N4—H2N4	113.3 (14)	C18—C17—H17B	109.2
H1N4—N4—H2N4	107 (2)	H17A—C17—H17B	107.9
C24—N4—H3N4	102.2 (17)	O6—C18—C17	108.55 (14)
H1N4—N4—H3N4	117 (2)	O6—C18—H18A	110.0
H2N4—N4—H3N4	109 (2)	C17—C18—H18A	110.0
O3—C1—N1	124.30 (16)	O6—C18—H18B	110.0
O3—C1—C2	121.76 (15)	C17—C18—H18B	110.0
N1—C1—C2	113.94 (14)	H18A—C18—H18B	108.4
N2—C2—C1	104.42 (13)	O6—C19—C20	109.31 (15)
N2—C2—H2A	110.9	O6—C19—H19A	109.8
C1—C2—H2A	110.9	C20—C19—H19A	109.8
N2—C2—H2B	110.9	O6—C19—H19B	109.8
C1—C2—H2B	110.9	C20—C19—H19B	109.8
H2A—C2—H2B	108.9	H19A—C19—H19B	108.3
C8—C3—C4	119.87 (15)	O7—C20—C19	108.74 (15)
C8—C3—N2	118.94 (15)	O7—C20—H20A	109.9
C4—C3—N2	121.19 (15)	C19—C20—H20A	109.9
O4—C4—C5	125.49 (16)	O7—C20—H20B	109.9
O4—C4—C3	115.86 (15)	C19—C20—H20B	109.9
C5—C4—C3	118.65 (15)	H20A—C20—H20B	108.3
C6—C5—C4	120.62 (16)	O7—C21—C22	109.08 (18)
C6—C5—H5	119.7	O7—C21—H21A	109.9
C4—C5—H5	119.7	C22—C21—H21A	109.9
C5—C6—C7	121.50 (16)	O7—C21—H21B	109.9
C5—C6—H6	119.3	C22—C21—H21B	109.9
C7—C6—H6	119.3	H21A—C21—H21B	108.3
C6—C7—C8	117.06 (15)	O8—C22—C21	112.5 (2)
C6—C7—C9	122.74 (15)	O8—C22—H22A	109.1
C8—C7—C9	120.11 (15)	C21—C22—H22A	109.1
C3—C8—C7	122.29 (16)	O8—C22—H22B	109.1
C3—C8—H8	118.9	C21—C22—H22B	109.1
C7—C8—H8	118.9	H22A—C22—H22B	107.8
C10—C9—C14	117.26 (16)	O8—C23—C24	111.71 (16)
C10—C9—C7	121.45 (15)	O8—C23—H23A	109.3
C14—C9—C7	121.26 (15)	C24—C23—H23A	109.3
C11—C10—C9	121.63 (16)	O8—C23—H23B	109.3
C11—C10—H10	119.2	C24—C23—H23B	109.3
C9—C10—H10	119.2	H23A—C23—H23B	107.9
C10—C11—C12	120.59 (16)	N4—C24—C23	109.41 (16)
C10—C11—H11	119.7	N4—C24—H24A	109.8
C12—C11—H11	119.7	C23—C24—H24A	109.8
C11—C12—C13	118.37 (16)	N4—C24—H24B	109.8
C11—C12—C16	123.98 (16)	C23—C24—H24B	109.8
C13—C12—C16	117.63 (16)	H24A—C24—H24B	108.2
C14—C13—C12	120.93 (17)		
			
O2—S1—N1—C1	121.10 (13)	C9—C7—C8—C3	175.66 (14)
O1—S1—N1—C1	−110.68 (13)	C6—C7—C9—C10	19.1 (2)
N2—S1—N1—C1	6.33 (14)	C8—C7—C9—C10	−157.39 (16)
O2—S1—N2—C3	68.27 (15)	C6—C7—C9—C14	−162.95 (16)
O1—S1—N2—C3	−58.24 (15)	C8—C7—C9—C14	20.6 (2)
N1—S1—N2—C3	−174.93 (14)	C14—C9—C10—C11	−1.7 (3)
O2—S1—N2—C2	−127.73 (12)	C7—C9—C10—C11	176.34 (15)
O1—S1—N2—C2	105.76 (13)	C9—C10—C11—C12	−1.1 (3)
N1—S1—N2—C2	−10.94 (13)	C10—C11—C12—C13	2.2 (2)
S1—N1—C1—O3	179.31 (14)	C10—C11—C12—C16	−176.56 (15)
S1—N1—C1—C2	0.00 (19)	C11—C12—C13—C14	−0.6 (3)
C3—N2—C2—C1	174.45 (15)	C16—C12—C13—C14	178.28 (17)
S1—N2—C2—C1	11.42 (16)	C12—C13—C14—C9	−2.3 (3)
O3—C1—C2—N2	173.35 (16)	C10—C9—C14—C13	3.4 (3)
N1—C1—C2—N2	−7.3 (2)	C7—C9—C14—C13	−174.70 (17)
C2—N2—C3—C8	127.60 (17)	C17—N3—C16—O5	7.3 (3)
S1—N2—C3—C8	−70.86 (19)	C17—N3—C16—C12	−173.40 (14)
C2—N2—C3—C4	−52.4 (2)	C11—C12—C16—O5	151.68 (17)
S1—N2—C3—C4	109.13 (16)	C13—C12—C16—O5	−27.1 (2)
C15—O4—C4—C5	−3.1 (2)	C11—C12—C16—N3	−27.6 (2)
C15—O4—C4—C3	177.13 (15)	C13—C12—C16—N3	153.56 (16)
C8—C3—C4—O4	−179.96 (14)	C16—N3—C17—C18	105.25 (19)
N2—C3—C4—O4	0.1 (2)	C19—O6—C18—C17	157.75 (15)
C8—C3—C4—C5	0.3 (2)	N3—C17—C18—O6	−59.8 (2)
N2—C3—C4—C5	−179.71 (15)	C18—O6—C19—C20	−159.42 (15)
O4—C4—C5—C6	−179.90 (15)	C21—O7—C20—C19	176.42 (17)
C3—C4—C5—C6	−0.2 (2)	O6—C19—C20—O7	70.69 (19)
C4—C5—C6—C7	−0.6 (3)	C20—O7—C21—C22	176.10 (19)
C5—C6—C7—C8	1.1 (2)	C23—O8—C22—C21	−119.2 (2)
C5—C6—C7—C9	−175.45 (15)	O7—C21—C22—O8	69.3 (3)
C4—C3—C8—C7	0.3 (2)	C22—O8—C23—C24	78.5 (2)
N2—C3—C8—C7	−179.69 (14)	O8—C23—C24—N4	55.8 (2)
C6—C7—C8—C3	−1.0 (2)		

### Hydrogen-bond geometry (Å, º)

Cg1 is the centroid of the C3–C8 ring.

**Table:**

*D*—H···*A*	*D*—H	H···*A*	*D*···*A*	*D*—H···*A*
N3—H1*N*3···O3^i^	0.82 (2)	2.22 (3)	3.012 (2)	161 (2)
N4—H1*N*4···O1^ii^	0.93 (3)	2.29 (3)	3.010 (2)	133 (2)
N4—H1*N*4···O7	0.93 (3)	2.49 (3)	3.106 (2)	124 (2)
N4—H2*N*4···N1^i^	1.03 (3)	1.82 (3)	2.821 (2)	163 (2)
N4—H3*N*4···O6^iii^	0.98 (3)	1.99 (3)	2.942 (2)	162 (3)
C2—H2*B*···O3^iv^	0.99	2.30	3.267 (2)	166
C18—H18*A*···N1^i^	0.99	2.57	3.545 (2)	168
C22—H22*A*···O8^ii^	0.99	2.63	3.343 (3)	129
C24—H24*A*···O5^iii^	0.99	2.58	3.298 (2)	129
C21—H21*B*···*Cg*1^ii^	0.99	2.70	3.555 (2)	165

Symmetry codes: (i) *x*, *y*, *z*+1; (ii) −*x*+1, −*y*+1, −*z*+1; (iii) *x*+1, *y*, *z*; (iv) −*x*+1, −*y*+2, −*z*.
